# Landscape and Spectrum of VWF Variants in Type 2 Von Willebrand Disease: Insights from a German Patient Cohort

**DOI:** 10.1055/a-2616-5161

**Published:** 2025-06-05

**Authors:** Hamideh Yadegari, Susan Halimeh, Alexander Krahforst, Anna Pavlova, Behnaz Pezeshkpoor, Jens Müller, Bernd Pötzsch, Arijit Biswas, Natascha Marquardt, Ute Scholz, Heinrich Richter, Heiner Trobisch, Karin Liebscher, Martin Olivieri, Karolin Trautmann-Grill, Oliver Tiebel, Ralf Knöfler, Johannes Oldenburg

**Affiliations:** 1 Institute of Experimental Haematology and Transfusion Medicine, Medical Faculty, University of Bonn, Bonn, Germany; 2 Coagulation Center Rhein-Ruhr, Duisburg, Germany; 3 Center of Hemostasis, MVZ Labor Leipzig, Leipzig, Germany; 4 Münster Hemostasis Center, Münster, Germany; 5 Laboratory and Ambulance for Coagulation Disorders, Duisburg, Germany; 6 Institute of Transfusion Medicine and Clinical Hemostaseology, Klinikum St. Georg GmbH, Leipzig, Germany; 7 Pediatric Thrombosis and Hemostasis Unit, Dr. Von Hauner Children's Hospital, LMU Clinic, Munich, Germany; 8 University Hospital Carl Gustav Carus, Technische Universität Dresden, Dresden, Germany; 9 Institute of Clinical Chemistry and Laboratory Medicine, University Hospital Carl Gustav Carus, Technische Universität Dresden, Dresden, Germany; 10 Pediatric Hemostaseology Unit, Department of Pediatrics, Faculty of Medicine and University Hospital Carl Gustav Carus, Technische Universität Dresden, Dresden, Germany

**Keywords:** von Willebrand disease, von Willebrand factor, type 2 von Willebrand disease, genetic variation

## Abstract

**Introduction:**

von Willebrand disease (VWD) type 2 arises from variants in von Willebrand factor (VWF) that disrupt its essential hemostatic functions. As per ISTH guidelines, it is classified as type 2A, 2B, 2M, and 2N based on the affected VWF roles.

**Objectives:**

This population-based study aims to uncover the genotype and laboratory phenotypes in type 2 VWD, providing insights into underlying genetics and genotype–phenotype associations.

**Patients/Methods:**

Our cohort included 247 patients from 196 families. Patients were characterized through multiple VWF phenotypic assays and genetic analyses, including DNA sequencing, copy number variation evaluations, and bioinformatic assessments.

**Results:**

A total of 86 index patients (IPs, 44%) were diagnosed with type 2A, the most prevalent subtype. Additionally, 27 IPs (14%) were diagnosed with type 2N, 24 IPs (12%) with type 2B, 17 IPs (9%) with type 2M, and 42 IPs categorized as type U VWD carried VWD-associated variants but could not be assigned to a specific subtype. VWF variants were detected in 187 out of 196 (95%) individuals. A total of 222 VWF variants were identified: 187 missense (84%), 22 null alleles (10%), 5 regulatory (2%), 6 gene conversions (3%), and 2 silent variants (1%). Many variants were recurrent in our cohort, resulting in 114 distinct variants. Of these, 45 (39%) were novel.

**Conclusion:**

Our data expands the spectrum of disease-associated variants in VWF, including many newly identified variants. This provides valuable insights for accurate diagnosis and personalized treatment. Additionally, the significant genetic heterogeneity among type 2 patients highlights the challenges in sub-classification.

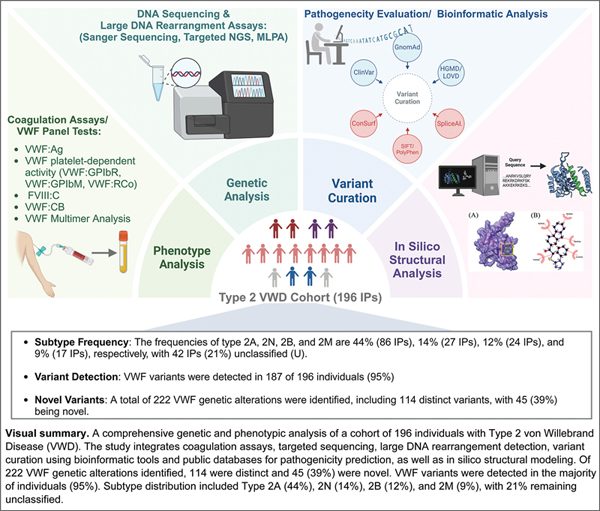

## Introduction

Von Willebrand disease (VWD) stands out as the most common hereditary bleeding disorder, arising from deficiencies or defects in the von Willebrand factor (VWF). 1 2 VWF, a large multimeric glycoprotein, plays a key role in hemostasis by facilitating platelet adhesion and aggregation at injury sites. 3 4 VWF multimers also protect and transport factor VIII (FVIII) to the site of injury. 5 6 The VWF gene ( *VWF* ) is positioned on chromosome 12p13.2, covering 178 kb and incorporating 52 exons. 7 *VWF* is translated to a pre-pro-VWF protein with a 741-aa propeptide (D1-D2 domains) and a 2050-aa mature subunit (comprising domains D'-D3-A1-A2-A3-D4-C1-C6-CK). 8 9 The D1, D2, D', D3, and C-terminal domains play pivotal roles in intracellular VWF biosynthesis, organizing processes such as dimerization and the assembly of high molecular weight multimers (HMWMs). 10 11 12 13 14 The A1, A2, A3, and D'-D3 domains contain binding sites for platelet GPIbα, collagen, and FVIII. Specifically, the A1 domain harbors the platelet glycoprotein Ibα (GPIbα) receptor binding site, the A3 domain encompasses binding sites for collagen types I and III, the A2 domain contains a cleavage site (Tyr1605-Met1606) for ADAMTS13 (a disintegrin and metalloprotease with thrombospondin-1 motifs #13), and the FVIII binding site is located in the D'-D3 domains. 15 16 17 18 19 Upon vessel injury, the extended conformation of VWF binds to exposed subendothelial collagen through A3 domain interactions with collagen types I and III. This exposure reveals the concealed platelet binding site to the GPIbα receptor in the A1 domain, initiating the formation of the platelet plug. 20 21 22 Additionally, VWF enhances aggregation through the C4 domain's interaction with GPIIb/IIIa. 23 ADAMTS13, in turn, regulates VWF multimer size and function. 24

According to the latest guidelines, VWD is grouped into three main types: type 1 (mild to moderate reduction of plasma VWF), type 2 (qualitative defects in VWF), and type 3 (complete absence of VWF). 25 26 27 28 29 Type 2 VWD is further divided into subcategories: 2A, 2B, 2M, and 2N. Type 2A is characterized by the loss or reduction of high molecular weight VWF multimers, resulting in diminished VWF binding functions. 28 30 Type 2B VWD arises from gain-of-function mutations in the A1 domain, resulting in increased affinity for platelet GPIbα, a deficit of multimers, and may be associated with thrombocytopenia under specific circumstances. 31 32 Type 2M is identified by reduced binding of VWF to platelet GPIbα or collagen. Notably, all types 2A, 2B, and 2M exhibit an autosomal dominant inheritance pattern. 33 Type 2N, on the other hand, is distinguished by reduced binding of FVIII to VWF and follows an autosomal recessive inheritance pattern. 34

In this study, we conducted a comprehensive evaluation of laboratory phenotypes and mutation patterns in a large cohort of individuals with type 2 VWD. Our aim was to advance our understanding of the molecular basis and pathophysiological mechanisms underlying qualitative VWD, shedding light on its crucial aspects. This investigation holds significance in guiding future therapeutic strategies and improving patient care.

## Method and Materials

### Cohort of Patients

A cohort comprising 575 patients from unrelated families suspected of having VWD was enrolled in year 2021. These patients were referred to the Bonn Haemophilia Center at the University Clinic Bonn, the Coagulation Center Rhein-Ruhr (GZRR) in Germany, and several other centers across the country. Patients were recruited based on a VWD diagnosis established by the treating physician and confirmed through laboratory tests in accordance with the updated ISTH-SSC VWF guidelines. 26 Inclusion criteria required patients to meet the diagnostic thresholds outlined in these guidelines, including specific VWF antigen levels and activity assays. The study primarily included individuals with inherited VWD, including both males and females. A small number of patients (5 out of 196) were confirmed to have acquired VWD after genetic testing and consultation with the treating physicians during the study. Although VWD has an autosomal inheritance pattern, women are more frequently diagnosed due to menstrual bleeding and bleeding after childbirth, leading to their overrepresentation in our cohort (approximately 60% female and 40% male, based on rough estimates). Following this analysis, 417 individuals were confirmed to have VWD. Among these, 196 index patients (IPs) were identified as having type 2 VWD, with their genotype and phenotype data detailed in this report. The patients were further subclassified based on laboratory phenotypes as well as genetic analysis. 26 35 36 Additionally, 221 IPs were classified as having quantitative VWD (types 1 and 3), and their results were published elsewhere. 37

The current study was prospectively designed and approved by the local ethics committee (vote 091/09). Informed consent was obtained from all patients in accordance with the Declaration of Helsinki, and assent was obtained for enrolled children following the prevailing regulations in Germany.

Blood samples from IPs and their family members were collected using both sodium citrate and EDTA tubes.

### Laboratory Evaluations

All laboratory tests were conducted at the source clinic attended by the patient. These included measurements of VWF antigen (VWF:Ag), VWF platelet-dependent activity (VWF activity [VWF:Ac]) assessed via recombinant GPIb and ristocetin (VWF:GPIbR), gain-of-function mutated GPIb (VWF:GPIbM), or VWF ristocetin cofactor assay (VWF:RCo), FVIII clotting activity (FVIII:C), and VWF collagen (type I or III) binding (VWF:CB). The levels of VWF:Ag and VWF:GPIbM were determined using BCS XP or Atellica Coag 360 coagulation analyzer (Siemens Healthcare, Germany) or by applying HemosIL AcuStar VWF:Ag and vWF:GP1bR assays (Instrumentation Laboratory, Werfen, Germany) following the manufacturer's instructions. The VWF:RCo was performed using in-house aggregometry-based assays, as detailed elsewhere. 38 VWF:CB was determined using enzyme-linked immunosorbent assays (Technoclone, Austria; or Hyphen Biomed, France) or the HemosIL AcuStar VWF:CB assay (Instrumentation Laboratory, Werfen, Germany) in accordance with the manufacturers' guidelines. The FVIII:C was assessed by various techniques, either using a one-stage clotting assay (OSCA), or chromogenic substrate assay (CSA), as described elsewhere. 39

The distribution of VWF multimers was determined through gel electrophoresis using 1.3 and 1.6% sodium dodecyl sulfate agarose gels, as previously outlined. 38 40

### DNA Analysis

Genetic analyses were performed at two laboratories, namely, the Department of Molecular Haemostaseology at the University Hospital Bonn and the GZRR center. Peripheral blood was used to isolate genomic DNA employing the Blood Core Kit (Qiagen, Germany). Sequence variations in the *VWF* were identified through direct sequencing, while the detection of large rearrangements utilized the multiplex ligation-dependent probe amplification (MLPA) technique or assessment of copy number variations (CNVs). Sanger sequencing methodology was applied for *VWF* analysis in patients investigated prior to 2016 (approximately 14% of IPs), whereas next-generation sequencing (NGS) was employed for the analysis of *VWF* in patients inspected from 2017 onwards (approximately 86% of IPs). In both strategies, the entire *VWF* , encompassing all 52 exons, intron/exon boundaries, and promoter regions, was analyzed.

#### Sanger Sequencing

The sequencing of the entire *VWF* was conducted using an ABI Prism 3130 genetic analyzer (Applied Biosystems by Life Technologies, Germany). 38 41 Subsequently, the generated sequences were aligned against the reference sequence using SeqScape Version 2.7 (Applied Biosystems by Life Technologies, Germany) to identify variations in the *VWF* .

#### Targeted NGS Analysis

Mini-Seq genome sequencer (Illumina, USA) was employed for the targeted NGS analysis, and the data were assessed by SeqPilot tool (JSI Medical Systems, Ettenheim, Germany). All detected variants were additionally verified by Sanger sequencing.

#### Large DNA Rearrangements Analysis

In cases where DNA sequencing failed to reveal any VWF variation, an additional analysis was performed to detect large deletions and duplications using MLPA and CNV evaluation. The SALSA MLPA kits (MRC-Holland, Netherlands) were utilized following the manufacturer's recommendations, and dosages were analyzed using Coffalyser (V5.2) software (MRC-Holland, Netherlands). The evaluation of CNVs was performed through SeqPilot (JSI Medical Systems GmbH). 42

#### Variant Curation

The pathogenicity of *VWF* variants was assessed based on the established criteria of the American College of Medical Genetics (ACMG) and the Association for Molecular Pathology (AMP). 43 To confirm the novelty of the identified VWF variants, a thorough cross-referencing was conducted against various comprehensive databases and published literature. These references encompassed disease-specific databases such as the Human Gene Mutation Database (HGMD, https://www.hgmd.cf.ac.uk/ac/index.php ) and the Leiden Open Variation Database (LOVD, https://databases.lovd.nl/shared/genes ). Additionally, searches were extended to population databases, including the ClinVar ( https://www.ncbi.nlm.nih.gov/clinvar/ ) and the Genome Aggregation Database (gnomAD, https://gnomad.broadinstitute.org/ ). These searches were executed in May 2023. 44 45 DNA variations were considered potential pathogenic candidates if their minor allele frequency (MAF) was found to be below 1% in the population databases.

### Bioinformatic Prediction Assessment

To assess the potential impact of missense and splice variants on VWF structure and function, the online tool Ensembl Variant Effect Predictor (VEP) was employed ( https://www.ensembl.org/info/docs/tools/vep/index.html , accessed in May 2024). VEP integrates bioinformatic prediction tools, including Polymorphism Phenotyping-2 (PolyPhen-2), Sorting Intolerant From Tolerant (SIFT), Mutation Taster, and Splice Artificial Intelligence (SpliceAI). 46 47 48 49 50 51 52 The bioinformatic tool ConSurf ( https://consurf.tau.ac.il/consurf_index.php , accessed in April 2022) was additionally employed to determine the evolutionary conservation of nucleic acid positions at the DNA level.

### In Silico Structural Analysis of Novel Variants

We conducted a structural analysis of novel VWF variants across the A1, A2, D2, D3, A3, and C4 domains using relevant Protein Data Bank (PDB) structures to assess their impact on stability, multimerization, and binding functions. For the A1 domain, we used PDB: 1AUQ and PDB: 1SQ0 to analyze its interaction with GPIbα and overall domain stability. 53 54 A2 and A3 variants were examined using PDB: 3GXB and PDB: 4DMU, respectively. 55 56 Structural insights into the D2 and D3 domain variants were obtained from PDB IDs: 7ZWH and 6N29, investigating their impact on VWF multimerization and VWF tubule helical structures. 57 58 Since the C2 domain lacks a biophysical structure, the p.Cys2565Ser was analyzed on an AlphaFold 3 modeled structure of the C2 domain. 59 C4 domain variants were analyzed using PDB: 6FWN, focusing on structural integrity and interactions with platelet integrin αIIbβ3. 60 61

Visualization, structural analysis, and rendering were performed using YASARA View. 62

## Results

**Fig. 1:**
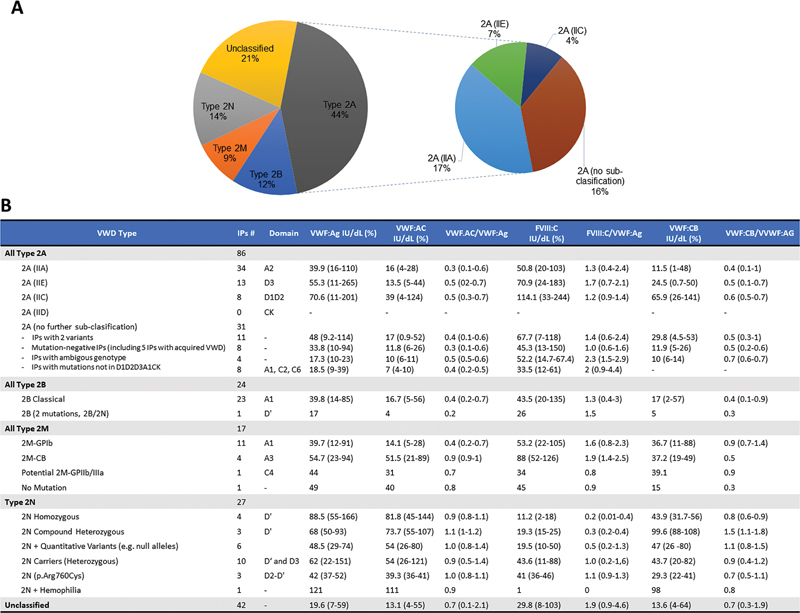
Subtype spectrum and summary of phenotype profile of 196 patients diagnosed with VWD type 2. ( **A** ) Frequency spectrum of type 2 VWD subtypes in the current cohort. Type 2A was the most common subtype (44%), followed by 2N (14%), 2B (12%), and 2M (9%). Additionally, 21% of patients were not categorized into the defined subtypes and are indicated as unclassified (U, 21%). ( **B** ) Summary of subtype composition along with the location of detected VWF variants and laboratory phenotype profile for each subtype of type 2 VWD presented in this cohort. For all presented laboratory parameters—VWF:Ag (IU/dL; %), VWF:AC (IU/dL; %), VWF:Ac/VWF:Ag ratio, FVIII:C (IU/dL; %), FVIII:C/VWF:Ag ratio, VWF:CB (IU/dL; %), and VWF:CB/VWF:Ag ratio—values are reported as the mean, with the lowest and highest values indicated in parentheses. For subcategories represented by only a single index patient (e.g., type 2B, and cases with dual mutations 2B/2N), individual values are provided instead of a range. The type 2A subtype is intricately subdivided into IIA, IIC, IID, and IIE based on distinct underlying pathologic mechanisms. IIA is caused by increased proteolysis by ADAMTS13 in the A2 domain, IIC is linked to multimerization defects from mutations in D1-D2 domains, IID results from mutations affecting dimerization in the cystine knot (CK), and IIE is a consequence of impaired VWF multimerization due to mutations occurring in the D3 domain. %, IU/dL; FVIII:C, FVIII coagulant activities; IPs, index patients; VWD, von Willebrand disease; VWF, von Willebrand factor; VWF:Ac, VWF binding activity to platelet GPIb; VWF:Ag, VWF antigen; VWF:CB, VWF binding activity to collagen.

Our current cohort of type 2 VWD comprised 196 IPs, totaling 247 patients including family members. Following ISTH-SSC guidelines, this subgroup includes 86 IPs classified as type 2A (44%), 27 IPs as type 2N (14%), 24 IPs as type 2B (12%), 17 IPs as type 2M (9%), and an additional 42 IPs (21%) demonstrating qualitative deficiencies in VWF with an unclear phenotype that could not be easily classified into one of the former subclasses; hence, they were considered unclassified (U) ( Fig. 1A ). At the time of recruitment for the current study, the cohort's demographic analysis revealed mean ages of 36.58 years (95% CI: 31.85 to 41.30 years) for type 2A, 41.38 years (95% CI: 31.36 to 51.40 years) for type 2B, 36.62 years (95% CI: 26.07 to 47.18 years) for type 2M VWD, 38.68 years (95% CI: 33.42 to 43.93 years) for the type 2N VWD sub-cohort, and 38.36 years (95% CI: 31.19 to 45.52 years) for the individuals with unclassified VWD type (type U). The putative mutations were identified in 187 out of 196 type 2 VWD individuals (approximately 95%). In total, 222 VWF variants (counting 114 distinctive variants) were identified in the type 2 VWD cohort, the majority of which were missense variants (84%).

### VWF Variant Spectrum and Phenotypic Laboratory Profiles in Type 2A VWD

Our type 2A VWD cohort consists of 116 patients from 86 families, displaying VWF:Ac/VWF:Ag ratio ranging from 0.1 to 0.7, with a mean of 0.40 ± 0.02 ( Fig. 1B ), along with impaired VWF multimers where multimer analysis was available (available for nearly 70% of IPs).

**Fig. 2:**
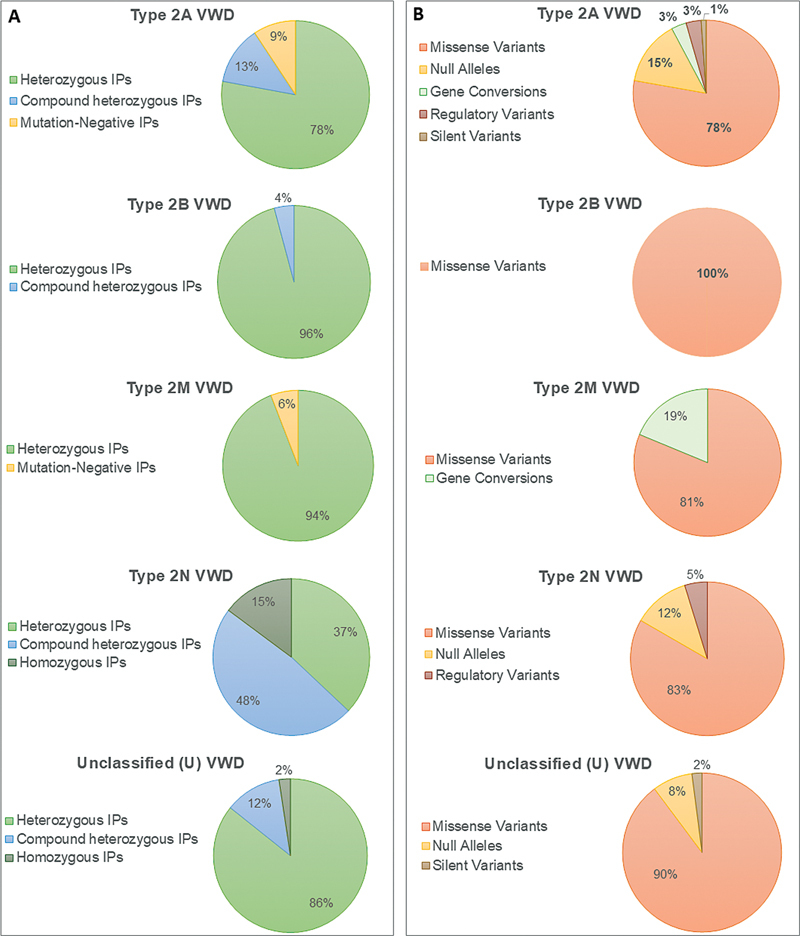
Mutation detection rate, zygosity pattern, and frequency spectrum of VWF variants in type 2 subtype cohorts. ( **A** ) The pie charts illustrate the success rate of mutation detection in each sub-cohort, including types 2A, 2B, 2M, 2N, and unclassified (U). ( **B** ) The pie charts display the spectrum of variant types, including missense variants, null alleles, gene conversions, silent variants, and promoter variants identified in each VWD subtype (2A, 2B, 2M, 2N, and U). In type 2A, null alleles encompassed three small deletions, three small duplications, two splice site variations, two large deletions, two small insertions, and one deletion/insertion. IPs, index patients; VWD, von Willebrand disease.

VWF variants were identified in 91% of individuals (78/86). A total of 91 variants, including 64 distinct variations, were detected. These included 70 missense variations (approximately 78%), 13 variants leading to null alleles (approximately 15%), 3 promoter variants (approximately 3%, all detected as the second variant), 3 gene conversions (approximately 3%), and 1 silent variant (approximately 1%) ( Fig. 2A ).

**Fig. 3:**
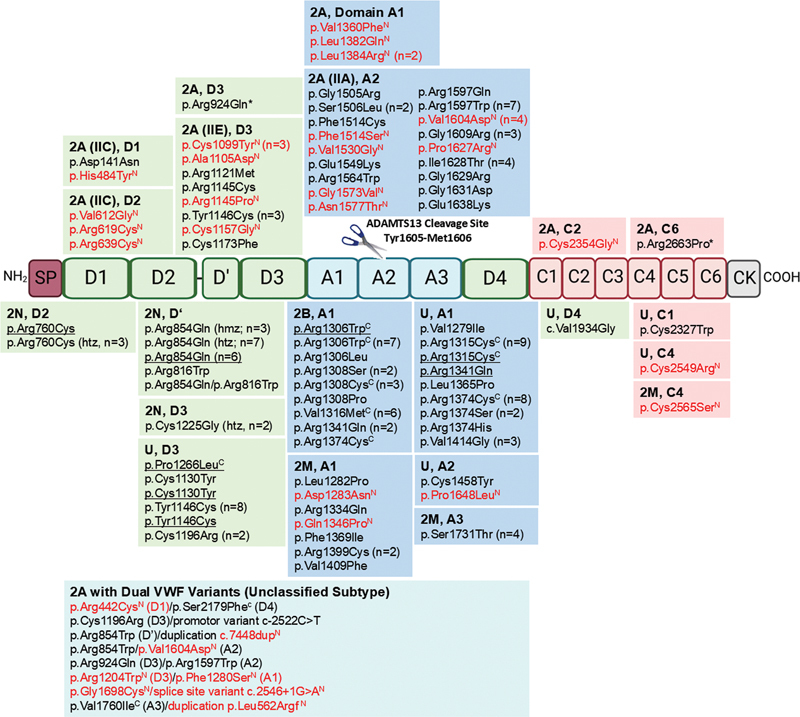
Schematic representation of the von Willebrand factor (VWF) protein structure and distribution of VWF variants detected in a cohort of type 2 von Willebrand disease (VWD). The schematic illustrates the domain organization of the VWF protein, including domains D1, D2, D', D3, A1, A2, A3, D4, C1, C2, C3, C4, C5, C6, and CK. VWF variants identified in patients with type 2 VWD (subtypes 2A, 2B, 2M, 2N, and unclassified [U]) are mapped to their corresponding domains. (1) Novel VWF variants are displayed in red and marked with a superscript N. (2) Variants detected in multiple patients are followed by a lowercase n in parentheses indicating the number of patients carrying that variant (e.g., ( *n* = 3)). (3) A superscript C indicates variants associated with accelerated VWF clearance. (4) A star (*) denotes variants that cannot fully explain the patient's phenotype, as these are linked to quantitative VWF deficiency rather than type 2 VWD, suggesting an ambiguous genotype. (5) Compound heterozygous variants are underlined. (6) For type 2N VWD, variants are labeled as homozygous (hmz), heterozygous (htz), or compound heterozygous (underlined). (7) Type 2A generally follows a dominant inheritance pattern, but some patients in our cohort carry more than one variant. Missense variants detected in these patients are presented in a blue-green box and categorized as type 2A with dual variants without further subclassification.

Beyond the ISTH classification, type 2A VWD can be further divided into four subgroups (IIA, IIC, IID, and IIE), each reflecting different molecular mechanisms that impair multimer elongation. 30 63 64 The classification into these subgroups is primarily based on the VWF domain containing the variant and is further confirmed by multimer analysis, when available. In our cohort, 34 of 86 individuals were classified as type 2A/IIA, with A2 domain variants that may induce increased VWF proteolysis by ADAMTS13. Common variants in this group included p.Arg1597Trp (seven IPs), p.Val1604Asp (four IPs), p.Ile1628Thr (four IPs), p.Gly1609Arg (three IPs), and p.Ser1506Leu (two IPs) ( Table 1.I ) ( Fig. 3 ). Most patients showed a lack of HMWMs and increased intensity in the side bands of the triplet structures. A total of 13 individuals were classified as type 2A/IIE, with D3 domain variants likely disrupting VWF alignment for multimerization. In this group, predominant variants involved missense mutations affecting cysteine residues in the D3 domain, accounting for 9 out of 13 IPs. These included p.Cys1099Tyr (three IPs), p.Cys1157Gly (one IP), and p.Cys1173Phe (one IP), or the introduction of cysteine, such as Tyr1146Cys (three IPs) and p.Arg1145Cys (one IP) ( Table 1.I ) ( Fig. 3 ). Eight individuals were identified as type 2A/IIC, with variants in the VWF propeptide, D1, and D2 domains, impairing multimerization. These included missense variants (five IPs), small deletions (two IPs), and a splice site variant (one IP) ( Table 1.I ). The remaining 31 type 2A individuals were not sub-classified. This group included patients with VWF variants located in the A1 or C domains (eight IPs), those with no identified VWF variant (eight IPs), multiple variants (11 IPs) ( Table 1.II ), or those with ambiguous genotypes (four IPs), such as p.Arg924Gln and gene conversions, where the phenotype could not be clearly explained ( Fig. 1B )( Table 1.I ). Upon further investigation and communication with the treating physicians, five of the eight mutation-negative IPs were later confirmed to have acquired VWD. Genetic analysis was performed for these patients to exclude pathogenic variants in the VWF gene.

**Table 1:** Detailed genotypic and phenotypic characteristics in type 2A VWD sub-cohort

**I. Type 2A patients with detected single VWF variant, constituting majority of index patients: 67 out of 86, along with mutation-negative index patients: 8 out of 86**											
IP #	Subtype	Nt change	aa change	Exon/Intron	Domain	Zygosity	Mutation type	Blood group	VWF:Ag (IU/dL)	VWF:Ac (IU/dL)	FVIII:C (IU/dL)
1	Type 2A (IIC)	c.421G > A	p.Asp141Asn	5	D1-VWD1	htz	Missense	na	11.4	4	na
2	Type 2A (IIC)	c.1309_1326del	p.Asp437_Arg442del	12	D2-VWD2	htz	Small Del.	A	201	124	244 ^CSA^
3	Type 2A (IIC)	c.1110–6_1110–5insC ^N^	–	-/9	D1-E1	htz	Splice Site	na	49	na	na
4	Type 2A (IIC)	c.1309_1326del	p.Asp437_Arg442del	12	D2-VWD2	htz	Small Del.	A	117	52	104 ^CSA^
5	Type 2A (IIC)	c.1450C > T ^N^	p.His484Tyr	13	D1-C8–1	htz	Missense	na	27	7	na
6	Type 2A (IIC)	c.1835T > G ^N^	p.Val612Gly	15	D2-C8–2	hmz	Missense	O	63	39 ^RCo^	88
7	Type 2A (IIC)	c.1855C > T ^N^	p.Arg619Cys	15	D2-C8–2	htz	Missense	A	28	14	33 ^CSA^
8	Type 2A (IIC)	c.1915C > T ^N^	p.Arg639Cys	15	D2-C8–2	htz	Missense	O	68	49	76 ^OSCA^
9	Type 2A	c.2771G > A*	p.Arg924Gln	21	D3-VWD3	htz	Missense	na	17	10	51
10–12	Type 2A (IIE)	c.3296G > A ^N^	p.Cys1099Tyr	25	D3-C8–3	htz	Missense	O/na/A	131/na/265	32/na/44	117 ^OSCA^ /na/183 ^CSA^
13	Type 2A (IIE)	c.3314C > A ^N^	p.Ala1105Asp	25	D3-C8–3	htz	Missense	na	23	na	45
14	Type 2A (IIE)	c.3362G > T	p.Arg1121Met	25	D3-C8–3	htz	Missense	A	11	5	21 ^CSA^
15	Type 2A (IIE)	c.3390C > T	p.Cys1130Cys	26	D3-TIL3	htz	Silent	A	41	na	82 ^OSCA^
16	Type 2A (IIE)	c.3433C > T	p.Arg1145Cys	26	D3-C8–3	htz	Missense	na	65	39	78 ^CSA^
17	Type 2A (IIE)	c.3434G > C ^N^	p.Arg1145Pro	26	D3-TIL-3	htz	Missense	B	17	5 ^GPIbR^	22
18–20	Type 2A (IIE)	c.3437A > G	p.Tyr1146Cys	26	D3-TIL-3	htz	Missense	na/A/A	19.3 /12/29	9/5/18	30/24 ^CSA^ /na
21	Type 2A (IIE)	c.3469T > G ^N^	p.Cys1157Gly	26	D3-TIL-3	htz	Missense	na	27	22 ^RCo^	57
22	Type 2A (IIE)	c.3518G > T	p.Cys1173Phe	26	D3-TIL-3	htz	Missense	na	23	16	37 ^CSA^
23	Type 2A	c.3686T > G* c.3692A > C	p.Val1229Gly p.Asn1231Thr	28 28	D3-E3 D3-E3	htz htz	Conversion	O	19	9 ^RCo^	37 ^OSCA^
24	Type 2A	c.3967_3969del ^N^	p.Asp1323del	28	A1	htz	Small Del.	O	14	6 ^RCo^	12
25, 26	2A	c.4027A > G* c.4079T > C c.4105T > A c.4133C > T c.4135C > T	p.Ile1343Val p.Val1360Ala p.Phe1369Ile p.Ser1378Phe p.Arg1379Cys	28	A1	htz	Conversion	O/O	23/10	11 ^RCo^ /6	67 ^OSCA^ /14.7
27	Type 2A	c.4042_4062dup ^N^	p.Lys1348_Val1354dup	28	A1	htz	Small Dupl.	A	19	9 ^RCo^	24
28	Type 2A	c.4078G > T ^N^	p.Val1360Phe	28	A1	htz	Missense	A	39	7	61 ^CSA^
29	Type 2A	c.4145T > A ^N^	p.Leu1382Gln	28	A1	htz	Missense	na	19	10	35
30, 31	Type 2A	c.4151T > G ^N^	p.Leu1384Arg	28	A1	htz	Missense	na/na	15/14	6 ^RCo^ /6	28/na
32	Type 2A (IIA)	c.4292_4299delinsGGATC ^N^	p.Gln1431_Pro1433delinsArgIle	28	A2	htz	Del. + Ins.	O	40	24	61 ^CSA^
33	Type 2A (IIA)	c.4513G > A	p.Gly1505Arg	28	A2	htz	Missense	O	21	4	27 ^CSA^
34, 35	Type 2A (IIA)	c.4517C > T	p.Ser1506Leu	28	A2	htz	Missense	-/A	55/65	35 ^RCo^ /10 ^RCo^	60/24
36	Type 2A (IIA)	c.4541T > G	p.Phe1514Cys	28	A2	htz	Missense	O	40	16	51 ^CSA^
37	Type 2A (IIA)	c.4541T > C ^N^	p.Phe1514Ser	28	A2	htz	Missense	B	22	14	27 ^CSA^
38	Type 2A (IIA)	c.4589T > G ^N^	p.Val1530Gly	28	A2	htz	Missense	na	30	na	na
39	Type 2A (IIA)	c.4645G > A	p.Glu1549Lys	28	A2	htz	Missense	na	110	14	103 ^CSA^
40	Type 2A (IIA)	c.4690C > T	p.Arg1564Trp	28	A2	htz	Missense	A	46	31	57 ^CSA^
41	Type 2A (IIA)	c.4718G > T ^N^	p.Gly1573Val	28	A2	htz	Missense	B	56	28	70 ^CSA^
42	Type 2A (IIA)	c.4730A > C ^N^	p.Asn1577Thr	28	A2	htz	Missense	A	45	14.0	41 ^CSA^
43–49	Type 2A (IIA)	c.4789C > T	p.Arg1597Trp	28	A2	htz	Missense	A/na/A/ O/B/na/ O	22/30/26/ 64/24/16/ 56	13/11 ^RCo^ /9/ 16/6/6/ 15	29/47/30 ^CSA^ / 66 ^CSA^ /32 ^CSA^ /20 ^CSA^ / 47
50	Type 2A (IIA)	c.4790G > A	p.Arg1597Gln	28	A2	htz	Missense	na	na	11	na
51–54	Type 2A (IIA)	c.4811T > A ^N^	p.Val1604Asp	28	A2	htz	Missense	O/A/O/ O	27/24/35/ 32	8/9/11/ 7	55 ^CSA^ /57 ^CSA^ /62 ^CSA^ / 53 ^CSA^
55–57	Type 2A (IIA)	c.4825G > A	p.Gly1609Arg	28	A2	htz	Missense	na/B/O	71/46/53	17/12/15	na/60 ^CSA^ /46 ^CSA^
58	Type 2A (IIA)	c.4880C > G ^N^	p.Pro1627Arg	28	A2	htz	Missense	O	19	4	21 ^CSA^
59–62	Type 2A (IIA)	c.4883T > C	p.Ile1628Thr	28	A2	htz	Missense	na/A/O/ na	28.7/38/17/ 55	5/7/5/ 9	32/37 ^CSA^ /20 ^CSA^ / 87 ^CSA^
63	Type 2A (IIA)	c.4885G > A	p.Gly1629Arg	28	A2	htz	Missense	B	45	8 ^GPIbR^	36
64	Type 2A (IIA)	c.4892G > A	p.Gly1631Asp	28	A2	htz	Missense	na	34	9	42
65	Type 2A (IIA)	c.4912G > A	p.Glu1638Lys	28	A2	htz	Missense	na	33	15	na
66	Type 2A	c.7060T > G ^N^	p.Cys2354Gly	41	C2	htz	Missense	na	56	39	89
67	Type 2A	c.7988G > C*	p.Arg2663Pro	49	C6	htz	Missense	na	9,3	4	41
68–75	Type 2A	None	–	–	–	–	–	A/O/na/ na/A/na/ A/A	24/41/10/ 94/41/17/ 17/26	na/10/na/ 14 ^RCo^ /26 ^RCo^ /7/ 8/6	29/25/na/ 150/26/na/ 13/28 ^CSA^
**II. Type 2A patients carrying two VWF variants, comprising 11 out of 86 index patients**											
#	Subtype	Nt change	aa change	Exon/Intron	Domain	Zygosity	Mutation type	Blood group	VWF:Ag (IU/dL)	VWF:Ac (IU/dL)	FVIII:C (IU/dL)
1	Type 2A*	c.-1224G > A ^N^ c.-2522C > T	–	–	–	htz htz	Regulatory Regulatory	na	40	17	71 ^CSA^
2	Type 2A	c.-2522C > T c.3586T > C	p.Cys1196Arg	27	D3-TIL-3	htz htz	Regulatory Missense	A	14	8 ^RCo^	34
3	Type 2A	c.1324C > T ^N^ c.6536C > T	p.Arg442Cys p.Ser2179Phe ^C^	12 37	D2-VWD2 D4-C8–4	htz htz	Missense Missense	na	na	17	15
4	Type 2A	c.1681_1684dup ^N^ c.5278G > A	p.Leu562Argfs p.Val1760Ile ^C^	14 30	D2-VWD2 A3	htz htz	Small Dupl. Missense	na	64	39	104 ^OSCA^
5	Type 2A	c.1682_1729 + 33 ^N^ c.1722_1723insA ^N^	p.Arg575Thrfs*16	15 14	D2 D2-C8–2	htz htz	Insertion / Splice Site Small Ins.	na	90	52	107 ^OSCA^
6	Type 2A/2N	c.2560C > T c.7448dup ^N^	p.Arg854Trp p.Tyr2483*	20 44	D'-E' C3	htz htz	Missense Small Dupl.	na	9	1	7
7	Type 2A	c.2546 + 1G > A ^N^ c.5092G > T ^N^	p.Gly1698Cys	19 29	D' A3	htz htz	Splice Site Missense	O	27	16	31 ^CSA^
8	Type 2A /2N	c.2561G > A c.4811T > A ^N^	p.Arg854Gln p.Val1604Asp	20 28	D'-E' A2	htz htz	Missense Missense	A	16	8	12 ^CSA^
9	Type 2A	c.2771G > A c.4789 C > T	p.Arg924Gln Arg1597Trp	21 28	D3-VWD3 A2	htz htz	Missense Missense	A	80	26 ^RCo^	118 ^OSCA^
10	Type 2A	c.3610C > T ^N^ c.3839T > C ^N^	p.Arg1204Trp p.Phe1280Ser	27 28	D3-E3 A1	htz htz	Missense Missense	na	26	14	34 ^CSA^
11	Type 2A	Del. 8 ^N^ Del. 52 ^N^	–	8 52	D1 CK	htz htz	Large Del. Large Del.	A	114	27	64 ^CSA^

Abbreviations: aa, amino acid; C, indicating variants accelerating VWF clearance; Del, deletion; Dupl, duplication; hmz, homozygous; htz, heterozygous; N, novel variants; Nt, nucleotide.

Notes: The D domains exhibit a consistent architecture, featuring VW domains, C8 folds, TIL structures, and E modules. Notably, exceptions occur in the D' domain, which lacks VW/C8, and the D4 domain, which lacks E but includes D4N.

The assessment of VWF binding activity to platelet GPIb (VWF:Ac) predominantly relied on the VWF:GPIbM assay, which employs recombinant mutated GPIb (without ristocetin). However, in specific instances, as indicated in the table, it was assessed using VWF:GPIbR, a method employing recombinant wild-type GPIb in the presence of ristocetin or the VWF:RCo assay. For some patients, the FVIII coagulant activities (FVIII:C) values were determined through either the one-stage clotting assay (OSCA) or the chromogenic substrate assay (CSA). Unmarked FVIII:C values signify that they were assessed using the one-stage assay based on actin FS.

The term “na” indicates when the laboratory value was not available. For patients for whom VWF:Ac or VWF:Ag data were missing, classification was performed based on multimer analysis, collagen-binding assays, or both, further supported by genetic analysis. The exception was the patient carrying the p.Val1530Gly variant, for whom VWF binding assays and multimer analysis were unavailable. However, our in vitro protein expression analysis confirmed increased ADAMTS13 cleavage, supporting the classification as type 2A-IIA (unpublished data). The “*” indicates that the genotype of the patients carrying these variants remains ambiguous, as these variants are known for causing a quantitative deficiency of VWF and cannot explain the laboratory phenotype observed in these patients. It is possible that patients carry a second DNA variation outside the VWF coding region.

### VWF Variant Spectrum and Phenotypic Laboratory Profiles in 2B VWD

The type 2B VWD sub-cohort comprised of 26 patients, originating from 24 families. Type 2B VWD sub-cohort was characterized by low VWF activity to antigen ratios and loss or reduction of HMW VWF multimers. In accordance with the recommended diagnostic algorithm, the diagnosis of type 2B VWD was further confirmed through genetic testing. In our sub-cohort of patients with type 2B VWD, the VWF:Ac/VWF:Ag ratios ranged from 0.2 to 0.7, with a mean value of 0.40 ± 0.04 ( Fig. 1B ). Multimer analysis, available for most of the patients (more than 70% of IPs), of this sub-cohort of patients with type 2B VWD revealed a loss of large multimers.

In all 24 IPs diagnosed with type 2B VWD, VWF variants were identified ( Fig. 2A ). Recurrent mutations were observed in this group, resulting in the detection of only nine VWF variants, all of which were missense mutations ( Fig. 2B ). Notably, these included the recurrent variant p.Arg1306Trp (detected in eight individuals), p.Arg1306Leu, p.Arg1308Ser (in two individuals), p.Arg1308Cys (in three individuals), p.Arg1308Pro, p.Val1316Met (identified in six individuals), p.Arg1341Gln (in two individuals), and p.Arg1374Cys. Among these type 2B VWD variants, the variants p.Arg1306Trp, p.Arg1308Cys, p.Val1316Met, and p.Arg1374Cys are also known for accelerating VWF clearance from the circulation ( Table 2 ) ( Fig. 3 ). 65 66

**Table 2:** Detailed genotypic and phenotypic characteristics in type 2B VWD sub-cohort

IP #	Nt change	aa change	Exon	Domain	Zygosity	Mutation type	Blood group	VWF:Ag (IU/dL)	VWF:Ac (IU/dL)	FVIII:C (IU/dL)
1	c.2561G > A c.3916C > T	p.Arg854Gln p.Arg1306Trp ^C^	20 28	D'-E' A1	htz	Missense Missense	A	17	4	26 ^CSA^
2–8	c.3916C > T	p.Arg1306Trp ^C^	28	A1	htz	Missense	A/na/O/B/ O/O/A	21/37/48/44/ 22/22/27	13 ^RCo^ /na/33/25/ 5/6/8	37/na/46/135 ^CSA^ / 27 ^CSA^ /20 ^CSA^ /33 ^CSA^
9	c.3917G > T	p.Arg1306Leu	28	A1	htz	Missense	A	85	56	30
10,11	c.3922C > A	p.Arg1308Ser	28	A1	htz	Missense	O/A	34/77	na/50	64 ^CSA^ /82 ^CSA^
12–14	c.3922C > T	p.Arg1308Cys ^C^	28	A1	htz	Missense	na /AB/A	42/30/14	15 ^RCo^ /6/10	48/41 ^CSA^ /26 ^CSA^
15	c.3923G > C	p.Arg1308Pro	28	A1	htz	Missense	B	26	5	25.0 ^CSA^
16–21	c.3946G > A	p.Val1316Met ^C^	28	A1	htz	Missense	B/O/A/na/ A/na	63/62/25/58/ 48/36	10/22 ^RCo^ /6/22/ 11/9	82 ^CSA^ /37/26/54/ 46 ^CSA^ /36 ^CSA^
22, 23	c.4022G > A	p.Arg1341Gln	28	A1	htz	Missense	A/O	42/32	17/15	51 ^CSA^ /55 ^CSA^
24	c.4120C > T	p.Arg1374Cys ^C^	28	A1	htz	Missense	na	19	8	na

Abbreviations: aa, amino acid; C, indicating variants accelerating VWF clearance; htz, heterozygous; N, novel variants; Nt, nucleotide.

Notes: The D domains exhibit a consistent architecture, featuring VW domains, C8 folds, TIL structures, and E modules. Notably, exceptions occur in the D' domain, which lacks VW/C8, and the D4 domain, which lacks E but includes D4N.

The evaluation of VWF binding activity to platelet GPIb (VWF:Ac) predominantly hinged on the VWF:GPIbM assay, performed with recombinant mutated GPIb and without ristocetin. However, in specific instances, as indicated in the table, it was assessed using the VWF:RCo assay. The measurement of FVIII coagulant activities (FVIII:C) was conducted using either one-stage assay based on actin FS (unmarked) or by chromogenic substrate assay (marked by CSA).

The term “na” indicates when the laboratory value was not available. For patients for whom VWF:Ac or VWF:Ag data were missing, classification was performed based on multimer analysis, collagen-binding assays, or both, further supported by genetic analysis.

Exceptionally, one of the individuals showed an additional variant known as a type 2N variant (p.Arg854Gln), in addition to the type 2B-specific VWF variant (p.Arg1306Trp), as compound heterozygous. This patient exhibited the lowest VWF:Ag (17%) and VWF:Ac (4%) levels, as well as an FVIII:C much lower than the average value of this sub-cohort (26%) ( Fig. 1B ).

### VWF Variant Spectrum and Phenotypic Laboratory Profiles in Type 2M VWD

The type 2M VWD sub-cohort comprised 23 patients from 17 families, characterized by diminished VWF activity to antigen ratios and normal or with ultra-large multimers ( Table 3 ). Several patients displayed VWF smeary multimer pattern. An exception was noted in a patient with a subtle loss of large multimers, characterized by a novel missense variant in domain A1 (p.Asp1283Asn), resulting in a very low VWF:Ac/VWF:Ag ratio (0.2) but a normal VWF:CB/VWF:Ag ratio (0.9).

**Table 3:** Detailed genotypic and phenotypic characteristics in type 2M VWD sub-cohort

IP #	Nt change	aa change	Exon	Domain	Zygosity	Mutation type	Blood group	VWF:Ag (IU/dL)	VWF:Ac (IU/dL)	FVIII:C (IU/dL)	VWF:CB (IU/dL)
1	c.3845T > C	p.Leu1282Pro	28	A1	htz	Missense	na	30	na	na	na
2	c.3847G > A ^N^	p.Asp1283Asn	28	A1	htz	Missense	A	45	9	70 ^CSA^	38
3	c.4001G > A	p.Arg1334Gln	28	A1	htz	Missense		41na44	na	na	na
4	c.4037A > C ^N^	p.Gln1346Pro	28	A1	htz	Missense	O	20	5	22 ^CSA^	20
5	c.4079T > C c.4105T > A c.4120C > T c.4133C > T	p.Val1360Ala p.Phe1369Ile p.Ser1378Phe p.Arg1374Cys	28	A1	htz	Conversion	B	23	11	39 ^CSA^	20
6, 7	c.4079T > C c.4105T > A c.4133C > T c.4135C > T	p.Val1360Ala p.Phe1369Ile p.Ser1378Phe p.Arg1379Cys	28	A1	htz	Conversion	O/O	52/12	18/8	44 ^CSA^ /27 ^CSA^	39/11
8	c.4105T > A	p.Phe1369Ile	28	A1	htz	Missense	na	30	16	61 ^CSA^	43
9, 10	c.4195C > T	p.Arg1399Cys	28	A1	htz	Missense	B/B	91/73	28/23	105 ^CSA^ /79 ^CSA^	88/57
11	c.4225G > T	p.Val1409Phe	28	A1	htz	Missense	O	21	9	32 ^CSA^	14
12–15*	c.5191T > A	p.Ser1731Thr	30	A3	htz	Missense	O/A/na/ na	23/47/94/na	21/46/89/50	52 ^CSA^ /86 ^CSA^ /126 ^CSA^ /na	na/na/49/25
16	c.7694G > A ^N^	p.Cys2565Ser	45	C4	htz	Missense	na	44	31	34 ^CSA^	39
17	None	–	–	–	–	–	na	49	40	45	15

Abbreviations: aa, amino acid; htz, heterozygous; N, novel variants; Nt, nucleotide; VWF:CB, von Willebrand factor collagen binding assay.

Notes: The measurement of FVIII coagulant activities (FVIII:C) was conducted using either one-stage assay based on actin FS (unmarked) or by chromogenic substrate assay (marked by CSA).

*Previous research related to the p.Ser1731Thr variant showed a significantly reduced binding to collagen type I, but a normal or only slightly decreased binding to collagen type III in static and flow-based VWF:CB assays, respectively. 33

The term “na” indicates when the laboratory value was not available. For patients for whom VWF:Ac and VWF:Ag data were missing, classification was performed based on multimer analysis, and supported by genetic analysis.

VWF variants were successfully identified in 16 out of 17 individuals (approximately 94%) ( Fig. 2A ). VWF variants in the A1 domain was found in 12 type 2M individuals, causing platelet-binding defects (2MGPIb subgroup) ( Fig. 1B ). The mean VWF:Ac/Ag and VWF:CB/VWF:Ag ratios were 0.39 ± 0.05 and 0.92 ± 0.07, respectively. This subgroup had eight distinct variants, including p.Leu1282Pro, p.Asp1283Asn, p.Arg1334Gln, p.Gln1346Pro, p.Phe1369Ile, p.Arg1399Cys (two IPs), p.Val1409Phe, and a gene conversion (three IPs) ( Table 3 ) ( Fig. 3 ). In four individuals, VWF variants in the A3 domain led to collagen-binding defects, forming the 2MCB subgroup, with p.Ser1731Thr as the common variant. 33 Additionally, one individual with a C4 domain variant (p.Cys2565Ser) showed a normal ratio profile but had ultra-large multimers, consistent with a type 2M phenotype ( Table 3 ).

### VWF Variant Spectrum and Phenotypic Laboratory Profiles in Type 2N VWD

Type 2N VWD, arising from mutations in the D'-D3 domains, interferes with VWF binding to FVIII and is inherited in a recessive manner. The diagnosis of suspected type 2N cases, in accordance with the ISTH-SSC VWF guidelines, were confirmed through DNA testing. In our cohort of type 2 VWD patients, 27 IPs were suspected of having type 2N and displayed type 2N-specific variants. Of these, seven were either homozygous or compound heterozygous for type 2N-specific VWF variants. Three individuals were homozygous for the most common type 2N VWF variant, p.Arg854Gln. Additionally, three individuals were compound heterozygous for the type 2N variants p.Arg816Trp and p.Arg854Gln, and one individual was homozygous for p.Arg816Trp. Within this subset, VWF:Ag and FVIII:C levels ranged from 50 to 166% and 2 to 25%, respectively, with a very low FVIII:C/VWF:Ag ratio ranging from 0.01 to 0.4 (mean 0.26 ± 0.05). Moreover, our cohort included individuals with compound heterozygosity for the common type 2N VWF variant p.Arg854Gln in combination with quantitative VWF variant (nonsense, small deletion, splice site variation, or the type 1-specific clearance variant p.Tyr1584Cys). 67 This group exhibited VWF:Ag and FVIII:C levels ranging from 29 to 74% and 10 to 50%, respectively, with an FVIII:C/VWF:Ag ratio ranging from 0.2 to 1.3 (mean 0.52 ± 0.16). Furthermore, within the type 2N subgroup, 10 individuals were identified as heterozygous for type 2N-specific variants, indicating a carrier state rather than a definitive diagnosis of type 2N VWD. Among them, eight individuals carried the heterozygous p.Arg854Gln variant—the most common type 2N VWF variant—while two carried the heterozygous p.Cys1225Gly variant. Since type 2N VWD follows a recessive inheritance pattern, a clinical diagnosis requires a homozygous or compound heterozygous state. Although these individuals were initially classified as having type 2N VWD, further analysis confirmed their heterozygous status, consistent with a carrier state. Notably, they exhibited a normal average FVIII:C/VWF:Ag ratio. Additionally, three individuals in the type 2N subgroup carried the heterozygous variant p.Arg760Cys, which affects the VWF propeptide cleavage site. 34 Furthermore, one patient with FVIII levels of 1% carried both a mutation in the F8 gene and the type 2N VWD variant p.Arg854Gln in VWF, explaining the extremely low FVIII levels despite unaffected VWF:Ag levels ( Fig. 2B ) ( Table 4 ).

**Table 4:** Detailed genotypic and phenotypic characteristics in type 2N VWD sub-cohort

IP #	Subtype	Nt change	aa change	Exon/Intron	Domain	Zygosity	Mutation type	Blood group	VWF:Ag (IU/dL)	VWF:Ac (IU/dL)	FVIII:C (IU/dL)
1	2N/1	c.-411T > C ^N^ c. + 167G > T c.2278C > T	- - p.Arg760Cys	- - 17	Regulatory Regulatory D2-E2	htz htz htz	Regulatory Regulatory Missense	na	37	36	46 ^CSA^
2	2N/1	c.1117C > T c.2561G > A	p.Arg373* p.Arg854Gln	10 20	D1-E1 D'-E'	htz htz	Nonsense Missense	B	43	na	10 ^OSCA^
3, 4	Heterozygous 2N	c.2278C > T	p.Arg760Cys	17	D2-E2	htz	Missense	na/na	52/37	41/ 41	na/36 ^CSA^
5, 6	2N/1	c.2435del c.2561G > A	p.Pro812Argfs*31 p.Arg854Gln	18 20	D'-TIL' D'-E'	htz htz	Small Del. Missense	na/na	29/74	26/80	10 ^CSA^ /40 ^CSA^
7	2N	c.2446C > T	p.Arg816Trp	19	D'TIL'	hmz	Missense	B	166	144	2
8–10	2N	c.2446C > T c.2561G > A	p.Arg816Trp p.Arg854Gln	19 20	D'-TIL' D'-E'	htz htz	Missense Missense	A/A/na	61/93/50	59/107/55	25 ^CSA^ /18 ^CSA^ /15 ^CSA^
11–13	2N	c.2561G > A	p.Arg854Gln	20	D'-E'	hmz	Missense	O/O/na	74/59/55	79/59/45	18/16/16 ^CSA^
14–21	Heterozygous 2N	c.2561G > A	p.Arg854Gln	20	D'-E'	htz	Missense	O/O/na/ na /A/A/ O/B	55/67/93/ 46/151/45/ 49 /22	30 ^Rco^ / 78 ^GPIbR^ /48/ 64/121/42/ 41/26	56 ^OSCA^ /88/76/ na/53 ^CSA^ /11 ^CSA^ / 23 ^CSA^ /53 ^CSA^
22	2N/1	c.2561G > A c.2649C > G ^N^	p.Arg854Gln p.Tyr883*	20 20	D'-E' D3-VWD3	htz htz	Missense Nonsense	na	63	60	18 ^CSA^
23	2N/1	c.2561G > A c.4751A > G	p.Arg854Gln p.Tyr1584Cys	20 28	D'-E' A2	htz htz	Missense Missense	A	40	54 ^GPIbR^	50
24	2N/1	c.2561G > A c.6798 + 2T > A ^N^	p.Arg.854Gln -	20 /38	D'-E' C1	htz htz	Missense Splice Site	AB	42	34	16
25	2N/Hemophilia A	c.2561G > A	p.Arg854Gln	20	D'-E'	htz	Missense	A	121	111 ^RCo^	1
26, 27	Heterozygous 2N	c.3673T > G	p.Cys1225Gly	27	D3-E3	htz	Missense	O/A	50/42	29/44	40 ^CSA^ /69 ^CSA^

Abbreviations: aa, amino acid; Del, deletion; hmz, homozygous; htz, heterozygous; N, novel variants; Nt, nucleotide.

Notes: The D domains exhibit a consistent architecture, featuring VW domains, C8 folds, TIL structures, and E modules. Notably, exceptions occur in the D' domain, which lacks VW/C8, and the D4 domain, which lacks E but includes D4N.

The assessment of VWF binding activity to platelet GPIb (VWF:Ac) predominantly relied on the VWF:GPIbM assay, which employs recombinant mutated GPIb (without ristocetin). However, in specific instances, as indicated in the table, it was assessed using VWF:GPIbR, a method employing recombinant wild-type GPIb in the presence of ristocetin or the VWF:RCo assay. For some patients, the FVIII coagulant activities (FVIII:C) values were determined through either the one-stage clotting assay (OSCA) or the chromogenic substrate assay (CSA). Unmarked FVIII:C values signify that they were assessed using the one-stage assay based on actin FS.

The term “na” indicates when the laboratory value was not available. For patients for whom VWF:Ac or VWF:Ag data were missing, classification was performed based on multimer analysis, collagen-binding assays, or both, further supported by genetic analysis.

### Genetic Variations in VWD Patients with Unclassified (U) Phenotype

In our cohort of qualitative VWD patients, we encountered 42 IPs who could not be definitively assigned to any specific VWD type. Consequently, they were classified as type U VWD. The ambiguity in classification arose from two primary factors. First, in many IPs within the type U group, detected mutations were consistently associated with different VWD types (type 2M, 2A, 1C, or even 2B) across various population-based studies, including our cohort. Two prominent examples are the VWF mutations p.Arg1315Cys and p.Arg1374Cys, identified in nine and eight IPs, respectively ( Fig. 3 ). Even within our cohort, IPs carrying these variants exhibited distinct phenotypic characteristics. For example, nine IPs with the variant p.Arg1315Cys displayed a wide range of VWF:Ac/VWF:Ag ratios, from 0.3 to 0.8. Second, in several IPs with novel VWF variants, a clear phenotype for precise VWD classification was elusive ( Table 5 ).

**Table 5:** Detailed genotypic and phenotypic characteristics in type U VWD subgroup

IP #	Subtype	Nt change	aa change	Exon/Intron	Domain	Zygosity	Mutation type	Blood group	VWF:Ag (IU/dL)	VWF:Ac (IU/dL)	FVIII:C (IU/dL)
1	U	c.2435del c.3797C > T	p.Pro812Argfs*31 p.Pro1266Leu ^C^	18 28	D'TIL' D3-E3	htz htz	Small Del. Missense	na	17	13 ^GPIbR^	49
2	U	C.2625C > T ^N^	p.Tyr875=	20	D3-VWD3	htz	Silent	O	17	15	18 ^CSA^
3	U (2A/2M/1C)	C.2771G > A c.3943C > T	p.Arg924Gln p.Arg1315Cys	21 28	D3-VWD3 A1	htz htz	Missense Missense	A	13	8	22 ^CSA^
4	U (2A/1)	c.3389G > A	p.Cys1130Tyr	26	D3-TIL-3	htz	Missense	na	14	14	23 ^CSA^
5	U (2A/1)	c.3389G > A c.3539–10T > G ^N^	p.Cys1130Tyr -	26 26	D3-TIL-3 D3-TIL-3	htz htz	Missense Splice Site	na	17	16	20 ^CSA^
6–8	U (2A/1)	c.3437A > G	p.Tyr1146Cys	26	D3-TIL3	htz	Missense	A/na/na	17/17/17	17/35/26	22/35/71
9	U	c.3437A > G c.3539–10T > G ^N^	p.Tyr1146Cys -	26 25	D3-TIL-3 D3-TIL-3	htz htz	Missense Splice Site	O	12	10	21 ^CSA^
10, 11	U	c.3586T > C	p.Cys1196Arg	27	D3-TIL-3	htz	Missense	A/O	21/18	18/12 ^RCo^	55 ^OSCA^ /27
12	U (2M/2B/1)	c.3835G > A	p.Val1279Ile	28	A1	htz	Missense	na	7	na	8
13–21	U (2A/2M/1C)	c.3943C > T	p.Arg1315Cys ^C^	28	A1	htz	Missense	A/O/A/ na/AB/A/ O/A/A	12/20/20/ 40/12/17/ 13/10 /14	7/13 ^RCo^ /15/ 10/6/14/ 8/6/4	26 ^CSA^ /39/28 ^OSCA^ / na /26 ^CSA^ /26 ^CSA^ / 22 ^CSA^ /12 ^CSA^ /31 ^CSA^
22	U	c.4022G > A c.5801T > G	p.Arg1341Gln c.Val1934Gly	28 34	A1 D4	htz htz	Missense Missense	na	34	27 ^RCo^	95
23	U (2A/1/M)	c.4094T > C	p.Leu1365Pro	28	A1	hmz	Missense	O	12	4	14 ^CSA^
24–31	U (2A/2M/1C)	c.4120C > T	p.Arg1374Cys ^C^	28	A1	htz	Missense	A/A/A/ A/O/na/ O/A	22/ 22/23 / 18/11/21/ 20/32	10/8/6/ 12/4/9/ 11/14	40 ^CSA^ /26 ^CSA^ /23 ^CSA^ / 22 ^CSA^ /11 ^CSA^ /42 ^CSA^ / 27 ^CSA^ /43 ^CSA^
32	U	c.4120C > A	p.Arg1374Ser	28	A1	htz	Missense	A	11	9	17 ^CSA^
33	U	c.4120C > A	p.Arg1374Ser	28	A1	htz	Missense	O	11	4	10 ^CSA^
34	U (2A/2M/1C)	c.4121G > A	p.Arg1374His	28	A1	htz	Missense	A	19	6	38 ^CSA^
35–37	U (2A/2M)	c.4241T > G	p.Val1414Gly	28	A1	htz	Missense	AB/A/O	14/42/15	9/15/7	30 ^CSA^ /53 ^CSA^ /25 ^CSA^
38	U (2A/2B)	c.4373G > A	p.Cys1458Tyr	28	A2	htz	Missense		19	1 ^RCo^	27
39	U	c.4943C > T ^N^	p.Pro1648Leu	28	A2	htz	Missense	A	59	55	57
40	U	c.5312–3C > G ^N^	–	31	A3	htz	Splice Site	B	28	20	47
41	U (A/1)	c.6981T > G	p.Cys2327Trp	41	C1	htz	Missense	O	37	32	103 ^CSA^
42	U	c.7645T > C ^N^	p.Cys2549Arg	45	C4	htz	Missense	O	9	16	41 ^CSA^

Abbreviations: aa, amino acid; C, indicating variants accelerating VWF clearance; Del, deletion; hmz, homozygous; htz, heterozygous; N, novel variants; Nt, nucleotide.

Notes: The D domains exhibit a consistent architecture, featuring VW domains, C8 folds, TIL structures, and E modules. Notably, exceptions occur in the D' domain, which lacks VW/C8, and the D4 domain, which lacks E but includes D4N.

The assessment of VWF binding activity to platelet GPIb (VWF:Ac) predominantly relied on the VWF:GPIbM assay, which employs recombinant mutated GPIb (without ristocetin). However, in specific instances, as indicated in the table, it was assessed using VWF:GPIbR, a method employing recombinant wild-type GPIb in the presence of ristocetin or the VWF:RCo assay. For some patients, the FVIII coagulant activities (FVIII:C) values were determined through either the one-stage clotting assay (OSCA) or the chromogenic substrate assay (CSA). Unmarked FVIII:C values signify that they were assessed using the one-stage assay based on actin FS.

### Exploring the Pathogenic Potential of Novel Missense and Splice Site Variants via Bioinformatics

#### Evaluating the Pathogenicity of Novel Missense Variants through In Silico Analysis

In the current type 2 VWD cohort, we identified 45 novel VWF variants, including 27 missense variants, 15 null alleles, 1 silent variant, and 2 regulatory variants. Most of these novel variants were observed in type 2A VWD. The pathogenicity of the novel missense variants was evaluated using predictive bioinformatic tools such as SIFT, PolyPhen-2, MutationTaster, and ConSurf. Of the 27 novel missense variants detected, 22 were consistently predicted to be deleterious or damaging by SIFT, PolyPhen-2, and MutationTaster. Notably, these variants predominantly occupied conserved residues, as indicated by ConSurf. In contrast, five missense variants showed discrepancies between the predictive outcomes of the bioinformatics tools, with one or two tools suggesting pathogenic effects while another indicated a benign or tolerated outcome ( Table 6.I ).

**Table 6:** Pathogenicity assessment of novel missense and splice site variants in type 2 VWD patients via bioinformatics tools

**I. In silico pathogenicity prediction of novel candidate missense variants in type 2 VWD patients by bioinformatics tools**										
**#**	**Subtypes**	**Nt change**	**aa change**	**Domain**	**In silico analysis**				**Existing in databases**	
					**SIFT (Score)**	**PolyPhen-2 (Score)**	**Mutation Taster (Score)**	**ConSurf**	**HGMD/LOVD/Pubmed**	**gnomAD (Frequency)/ClinVar**
**1**	Type 2A	c.1324C > T	p.Arg442Cys	D1	Deleterious (0)	Probably damaging (0.997)	Disease causing (0.810)	8 (e,f)	No	Yes (3.10e-5)/VUS
**2**	Type 2A	c.1450C > T	p.His484Tyr	D1	Deleterious (0.01)	Benign (0.401)	Disease causing (0.357)	4 (e)	No	Yes (4.96e-6)/No
**3**	Type 2A	c.1835T > G	p.Val612Gly	D2	Deleterious (0)	Probably damaging (0.992)	Disease causing (0.810)	9 (b,s)	No	Yes (2.82e-6)/No
**4**	Type 2A	c.1855C > T	p.Arg619Cys	D2	Deleterious (0)	Probably damaging (0.997)	Disease causing (0.897)	4 (e)	No	Yes (5.85e-6)/No
**5**	Type 2A	c.1915C > T	p.Arg639Cys	D2	Tolerated (0.22)	Possibly damaging (0.659)	Polymorphism (0.212)	2 (b)	No	Yes (1.59e-5)/No
**6**	Type 2A	c.3296G > A	p.Cys1099Tyr	D3	Deleterious (0)	Probably damaging (0.99)	Disease causing (0.810)	9 (b,s)	No	No/No
**7**	Type 2A	c.3314C > A	p.Ala1105Asp	D3	Deleterious (0)	Probably damaging (0.995)	Disease causing (0.810)	9 (b,s)	No	No/No
**8**	Type 2A	c.3434G > C	p.Arg1145Pro	D3	Deleterious (0)	Probably damaging (0.986)	Disease causing (0.810)	9 (e,f)	No	No/No
**9**	Type 2A	c.3469T > G	p.Cys1157Gly	D3	Deleterious (0)	Probably damaging (0.968)	Disease causing (0.810)	9 (b,s)	No	No/No
**10**	Type 2A	c.3610C > T	p.Arg1204Trp	D3	Deleterious (0)	Probably damaging (0.972)	Polymorphism (0.308)	2 (e)	No	Yes (2.42e-5)/No
**11**	Type 2A	c.3839T > C	p.Phe1280Ser	A1	Deleterious (0)	Probably damaging (1)	Disease causing (0.810)	7 (b)	No	No/No
**12**	Type 2M	c.3847G > A	p.Asp1283Asn	A1	Deleterious (0)	Probably damaging (0.985)	Disease causing (0.810)	9 (e,f)	No	No/No
**13**	Type 2M	c.4037A > C	p.Gln1346Pro	A1	Deleterious (0.04)	Probably damaging (0.999)	Polymorphism (0.089)	1 (e)	No	No/No
**14**	Type 2A	c.4078G > T	p.Val1360Phe	A1	Deleterious (0)	Probably damaging (0.997)	Disease causing (0.536)	7 (b)	No	No/VUS
**15**	Type 2A	c.4145T > A	p.Leu1382Gln	A1	Deleterious (0)	Probably damaging (1)	Disease causing (0.462)	6 (b)	No	No/No
**16**	Type 2A	c.4151T > C	p.Leu1384Pro	A1	Deleterious (0)	Probably damaging (1)	Disease causing (0.810)	7 (b)	No	No/VUS
**17**	Type 2A	c.4541T > C	p.Phe1514Ser	A2	Deleterious (0)	Possibly damaging (0.994)	Disease causing (0.810)	9 (b,s)	No	No/No
**18**	Type 2A	c.4589T > G	p.Val1530Gly	A2	Deleterious (0.02)	Probably damaging (0.98)	Disease causing (0.432)	5 (e)	No	No/No
**19**	Type 2A	c.4718G > T	p.Gly1573Val	A2	Deleterious (0)	Probably damaging (1)	Disease causing (0.810)	9 (e,f)	No	No/No
**20**	Type 2A	c.4730A > C	p.Asn1577Thr	A2	Deleterious (0)	Benign (0.299)	Disease causing (0.536)	9 (e,f)	No	No/No
**21**	Type 2A	c.4811T > A	p.Val1604Asp	A2	Deleterious (0)	Probably damaging (0.976)	Disease causing (0.810)	9(b,s)	No	No/No
**22**	Type 2A	c.4880C > G	p.Pro1627Arg	A2	Deleterious (0)	Probably damaging (0.999)	Disease causing (0.810)	8 (b)	No	No/No
**23**	U	c.4943C > T	p.Pro1648Leu	A2	Deleterious (0.01)	Probably damaging (0.999)	Disease causing (0.810)	9 (e,f)	No	Yes (8.90e-6)/No
**24**	Type 2A	c.5092G > T	p.Gly1698Cys	A3	Deleterious (0)	Probably damaging (0.999)	Disease causing (0.587)	7 (e)	No	Yes (6.57e-6)/No
**25**	Type 2A	c.7060T > G	p.Cys2354Gly	C2	Deleterious (0)	Possibly damaging (0.883)	Disease causing (0.810)	9 (e,f)	No	Yes (1.59e-6)/No
**26**	U	c.7645T > C	p.Cys2549Arg	C4	Deleterious (0)	Probably damaging (0.963)	Disease causing (0.810)	9 (b,s)	No	No/No
**27**	Type 2M	c.7694G > A	p.Cys2565Ser	C4	Deleterious (0)	Probably damaging (0.963)	Disease causing (0.519)	9 (b,s)	No	No/No
**II. Pathogenicity evaluation for newly identified splice site variants using Illumina AI splicing prediction tool incorporated in Ensembl Variant Effect Predictor (VEP)**										
**#**		**Nt change**		**Phenotype**	**Illumina SpliceAI**					
					**Acceptor loss Δ Score**	**Acceptor gain Δ Score**	**Donor loss Δ Score**	**Donor gain Δ Score**		
**1**		**c.1110–6_1110–5insC**		**Type 2A**	**0.00**	**0.00**	**0.00**	**0.00**		
**2**		**c.2546 + 1G > A**		**Type 2A**	**0.00**	**0.00**	**0.83**	**0.03**		
**3**		**c.3539–10T > G**		**Type U**	**0.01**	**0.00**	**0.00**	**0.00**	
**4**		**c.5312–3C > G**		**Type U**	**0.63**	**0.04**	**0.00**	**0.00**		
**5**		**c.6798 + 2T > A**		**Type 2N**	**0.00**	**0.00**	**0.99**	**0.36**	

Abbreviations: aa, amino acid; Nt, nucleotide; VUS, variant of uncertain significance; VWF, von Willebrand factor.

Notes: I. To predict the potential impact of newly identified variants on protein function, we utilized several in silico prediction tools. SIFT (Sorting Intolerant From Tolerant) analyzes interspecific and evolutionary sequence conservation. PolyPhen-2 (Polymorphism Phenotyping-2) assesses structural parameters as differential criteria in its analyses. MutationTaster 2021 utilizes supervised machine learning analysis, incorporating computational, mathematical, and biochemical parameters for predictions. ConSurf employs a multi-alignment pipeline and conservation profile construction to assess conservation. SIFT categorizes variants as tolerated or deleterious substitutions, assigning a normalized probability score. Variants with scores <0.05 are predicted as deleterious, while those with scores ≥0.05 are considered tolerated. PolyPhen-2 generates predictions of probably damaging, possibly damaging, or benign outcomes, accompanied by a numerical score ranging from 0.0 (benign) to 1.0 (damaging). MutationTaster prediction scores range from 0 to 1, with higher scores indicating a higher likelihood of pathogenicity. Prediction categories include “disease_causing_automatic,” “disease causing,” “polymorphism,” and “polymorphism-automatic.” The score cutoff between “disease causing” and “polymorphism” is 0.317.

ConSurf provides a conservation score on a scale of 1 to 9, indicating the degree of conservation of the mutated residues' corresponding amino acids.

ConSurf conversation scale:

The presence of variants was evaluated by cross-referencing disease-specific databases such as HGMD (Human Mutation Gene Database), LOVD (Leiden Open Variation Database), and Pubmed (literature), alongside population databases like gnomAD and ClinVar. DNA variations were considered potential pathogenic candidates if their minor allele frequency (MAF) was found to be below 1% in the population databases.

II. The Illumina SpliceAl predicted the effect of variants on core splice sites by providing delta (Δ) scores ranging from 0 to 1. The suggested cutoffs are 0.2 (high recall), 0.5 (recommended), and 0.8 (high precision).

#### In Silico Evaluation of Splice Site Variant Pathogenicity

We identified five potential novel splice variants in type 2A (c.1110–6_1110–5insC and c.2546 + 1G > A), type 2N (c.6798 + 2T > A, coupled with a type 2N-specific variant), and type U (c.5312–3C > G and c.3539–10T > G). To assess the potential consequences of these splicing variants, we utilized the Illumina SpliceAI tool integrated into VEP. Our analysis indicated that variants located in core consensus splice site residues (c.2546 + 1G > A, c.6798 + 2T > A, and c.5312–3C > G) could impact splicing efficiency. However, two variants located outside the consensus donor splice sites (+ and −5 nucleotides), namely, c.1110–6_1110–5insC and c.3539–10T > G, did not exhibit any apparent impact on splicing efficiency according to the SpliceAI predictive tool ( Table 6.II ).

### In Silico Structural and Functional Consequences of Novel Variants

**Fig. 4:**
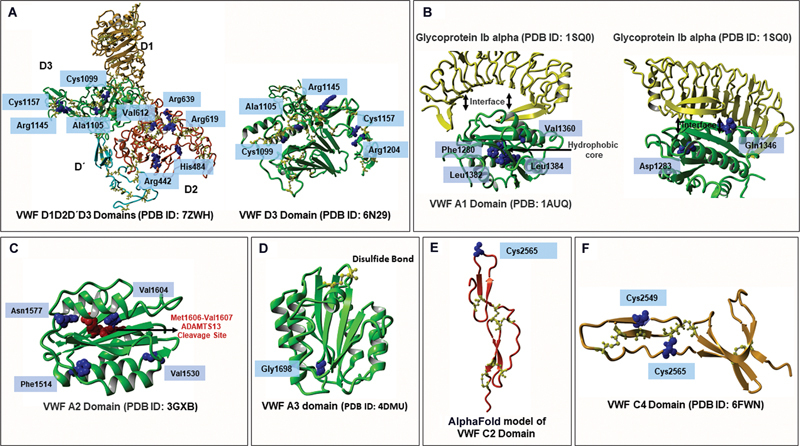
Structural and functional impact of novel VWF variants across key domains analyzed in silico. The impact of novel von Willebrand factor (VWF) variants on domain structure and function was analyzed in silico across domains D2, D3, A1, A2, A3, C2, and C4. Panels **A** to **F** depict the structural consequences of these variants. ( **A** ) D2 and D3 domains: Structural impacts were examined using PDB IDs: 7ZWH and 6N29. ( **B** ) A1 domain: Using PDB IDs: 1AUQ and 1SQ0, the effects on platelet GPIbα binding and domain stability were analyzed. ( **C** ) A2 domain: PDB: 3GXB was used to analyze A2 novel variants. ( **D** ) A3 domain: The A3 domain novel variants were analyzed using PDB: 4DMU. ( **E** ) C2 domain: Using an AlphaFold model, the p.Cys2354Gly variant was analyzed. ( **F** ) C4 domain: Structural consequences of C4 novel variants were assessed using PDB: 6FWN.

Novel variants within the D2 domain are predicted to cause significant destabilization, impairing multimer formation. The p.Arg442Cys variant disrupts electrostatic interactions and promotes non-native disulfide bond formation, while p.His484Tyr alters critical hydrogen bonding. The p.Val612Gly variant increases flexibility, destabilizing the hydrophobic core, and both p.Arg619Cys and p.Arg639Cys interfere with electrostatic interactions, leading to misfolding and abnormal disulfide bonding ( Fig. 4A ). In the D3 domain, variants such as p.Cys1099Tyr and p.Cys1157Gly disrupt essential disulfide bonds, while p.Ala1105Asp weakens hydrophobic interactions. The p.Arg1145Pro variant increases domain rigidity, and p.Arg1204Trp introduces steric clashes, collectively destabilizing the domain and impairing multimer formation ( Fig. 4A ).

In the A1 domain, mutations at residues Phe1280, Leu1382, Leu1384, and Val1360, while not directly involved in the GPIbα heterodimeric interface, significantly contribute to the domain's overall stability. Phe1280 and Leu1382 are critical for maintaining the hydrophobic core, while Leu1384 further reinforces structural integrity. Although Val1360 is positioned near the GPIbα interface, it aids in stabilizing the binding site's conformation through hydrophobic interactions without direct contact. Substitutions at these residues compromise VWF stability and multimerization by altering structural conformation. In contrast, the variants p.Asp1283Asn and p.Gln1346Pro directly impact platelet binding via GPIbα, with Asp1283 forming essential hydrogen bonds and electrostatic interactions that stabilize the A1–GPIbα interaction. Gln1346 also contributes to this interface, influencing the specificity and strength of binding ( Fig. 3B ). Novel variants in the A2 domain, including p.Phe1514Ser, p.Val1530Gly, p.Val1604Asp, p.Asn1577Thr, p.Pro1627Arg, and p.Pro1648Leu, destabilize the domain, increasing susceptibility to cleavage at the Tyr1605-Met1606 site. In contrast, p.Gly1573Val alters domain stability and flexibility without affecting cleavage susceptibility; this substitution introduces a bulkier side chain that may restrict movement and cause steric clashes, leading to localized rigidity that impairs domain stability and likely hinders proper VWF dimer alignment during multimerization ( Fig. 4C ). The p.Gly1698Cys variant in the A3 domain of VWF substitutes glycine with cysteine, introducing a larger, sulfur-containing side chain that increases the likelihood of non-native disulfide bond formation with adjacent cysteines. This alteration further compromises structural integrity and likely impairs the alignment of VWF dimers necessary for multimer elongation ( Fig. 3D ).

The p.Cys2354Gly variant in the C2 domain occurs at an unpaired cysteine. Although this cysteine is unpaired within the domain, it may participate in interdomain disulfide bonds. Consequently, the Cys2354Gly substitution could potentially disrupt key interdomain interactions, impairing VWF dimer formation and exerting a dominant-negative effect on VWF biosynthesis ( Fig. 4E ). Lastly, the p.Cys2549Arg and p.Cys2565Ser variants in the VWF C4 domain are predicted to disrupt the domain's structural integrity and platelet binding efficacy (interaction with platelet integrin αIIbβ3). Both Cys2549 and Cys2565 participate in critical disulfide bonding. The substitution of cysteine with arginine at position 2549 disrupts electrostatic balance, and the serine substitution at 2565 introduces weaker hydrogen bonding instead of strong disulfide linkages. These alterations disrupt critical disulfide bonds, destabilizing the domain and reducing platelet integrin (αIIbβ3) binding ( Fig. 3F ). 60 61

## Discussion

The current study represents one of the largest cohorts of type 2 VWD patients, examining the mutation spectrum and laboratory phenotype among 196 IPs in Germany, totaling 247 when including family members. Initially, our cohort comprised 417 IPs with both quantitative and qualitative VWD, including 221 IPs diagnosed with quantitative VWD (types 1 and 3) and 196 with type 2 VWD. The analysis of the quantitative cohort has been published elsewhere, 37 while this study focuses on the qualitative VWD (type 2) patients. 37

Among the current type 2 VWD cohort, 44% of patients had type 2A (the most common subtype), followed by 14% with type 2N, 12% with type 2B, 9% with type 2M, and 21% with unclassified VWD (type U). Within the type 2A group, subtype 2A/IIA was the most prevalent, accounting for 39%.

The distribution of type 2 VWD subtypes in our cohort follows the pattern of 2A > 2N > 2B > 2M. Notably, heterozygous carriers of type 2N-specific variants do not meet the diagnostic criteria for type 2N VWD, as this subtype follows a recessive inheritance pattern. Some initially diagnosed cases were later confirmed as carriers through genetic analysis. Similarly, type 2A was the most prevalent subtype in Spanish, US, Czech/ Slovak (referred to as the heart of Europe), and Chinese cohorts, as well as in our previously reported cohort from 2012. 38 68 69 70 71 72 However, in the French and Italian (Milan) cohorts, type 2M was the most prevalent, followed by type 2A. 73 74 In accordance with our cohort, subtype 2A/IIA emerged as the most common type 2A subgroup across Milan, France, and Czech/Slovak populations. 64 73 74 75

The updated guidelines recommended a VWF activity/VWF ratio cutoff of <0.7 to confirm type 2 VWD (2A, 2B, and 2M), a criterion we employed in our study. 26 Among type 2A patients, approximately 87% had a VWF activity/VWF ratio of ≤0.6, with four patients exhibiting a ratio of exactly 0.7. After comprehensive evaluations, including multimer analysis, VWF collagen binding assays, and genetic analysis, these patients were classified as type 2A. Similarly, over 90% of type 2B patients showed a VWF activity/VWF ratio of ≤0.6. Three patients with a ratio of 0.7 were categorized as type 2B based on reduced VWF HMWMs and the presence of specific variants. In the type 2M cohort, most patients had a VWF activity/VWF ratio of <0.6; however, six patients with ratios of ≥0.7 were noted, including index patients with mutations in the A3 domain that affect collagen binding. 33 In the present cohort of type 2 VWD, variants in the *VWF* were identified in 187 of the 196 individuals (95%). Mutation-negative cases were exclusively found within the type 2A sub-cohort (8 out of 86 individuals) and type 2M (1 out of 17 individuals). Notably, five of the mutation-negative cases within the type 2A cohort were later confirmed to have acquired VWD. Overall, 222 VWF variants were detected, comprising 114 distinct variations after eliminating duplicates. In type 2B and type 2M, all identified variants were missense mutations. Consistent with previous population-based studies, missense mutations accounted for the majority of variants in type 2A (78%), type 2N (83%), and subgroup U (90%). 38 73 74 Null alleles were predominantly identified in type 2A (13 out of 22), followed by type 2N (5 alleles) and subgroup U (4 alleles). Previous in vitro studies have elucidated the molecular mechanisms underlying VWF deletions and core splice site variants, which exhibit a dominant-negative effect, impeding the elongation of large VWF multimers and contributing to type 2A VWD. 76 77 78 In type 2N VWD, null alleles were identified alongside type 2N-specific variants (compound heterozygous), correlating with reduced plasma VWF levels compared with typical type 2N VWD patients, a finding consistent with reports from other cohorts. 34 79

Among the 114 distinct variants identified, 45 (39%) were reported for the first time in the VWD population, representing more than one-third of the total. Most of these novel variants were predicted to be pathogenic according to bioinformatics analyses. In silico structural predictions indicate that all these novel VWF variants may disrupt domain stability, hinder multimerization, and impair critical functional interactions essential for platelet adhesion. Each population-based study has identified a significant number of novel VWF variants, highlighting the genetic complexity and heterogeneous clinical presentation of this condition. 68 71 74 80

The challenge in classifying certain VWD patients as type U underscores the complexity of VWF structure–function relationships and the limitations of current diagnostic criteria. Several genetic variants, such as p.Tyr1146Cys, p.Arg1315C, p.Arg1374Cys, and p.Arg1374His, consistently appear in different VWD types (types 1, 2A, 2M, and 2B), both in the literature and our own cohort, creating ambiguity in phenotype determination. 72 81 82 We classified individuals with these variants as type U. These discrepancies may be influenced by multiple factors, including variability in plasma VWF levels (such as ABO blood type, age, pregnancy, medications, and underlying diseases), as well as variability in laboratory assays. Additionally, recent structural analyses by Seidizadeh et al have highlighted how the unique positions of these variants in the A1-A2 domains could disrupt both GPIb binding and multimerization. This dual impact might explain why some patients exhibit characteristics overlapping between types 2A and 2M, further complicating subtype classification. 83 In addition, the complexity of genotype–phenotype correlations in VWD is further illustrated by studies like that of van Kwawegen et al, which reported a strong association between the p.Arg1306Trp variant and thrombocytopenia in type 2B VWD. 84 Such genotype–phenotype correlations are valuable in understanding the clinical implications of specific variants and underscore the need for more comprehensive studies that integrate both genetic and clinical data. Additionally, in our cohort, some patients had inconclusive genotypes. This group included mutation-negative individuals and those with variants that did not fully explain their phenotypes, such as type 2A patients with VWF variants typically associated with quantitative deficiencies.

In summary, our study reveals novel molecular mechanisms and expands the spectrum of VWF variants, including newly identified mutations. By integrating phenotypic and genotypic data, we provide deeper insights into phenotype–genotype correlations in VWD. These findings enhance our understanding of the disease, improve diagnostic accuracy, and support personalized treatment. Additionally, the significant genetic heterogeneity and complex phenotypic variations in type 2 VWD underscore the challenges in precise classification, particularly where co-inherited mutations complicate diagnosis.

## Notes

**What is known about this topic?**

- Type 2 von Willebrand disease (VWD) arises from qualitative defects in von Willebrand factor (VWF) that impair its hemostatic functions.
- It is categorized into subtypes 2A, 2B, 2M, and 2N, each with distinct molecular mechanisms and clinical phenotypes.
- Genetic heterogeneity in type 2 VWD complicates diagnosis and classification.

**What does this paper add?**

- This study identifies 114 unique VWF variants, including 45 novel variants, significantly expanding the spectrum of disease-associated mutations.
- Findings highlight the challenges of genetic heterogeneity in type 2 VWD, emphasizing the need for refined diagnostic criteria.
- Comprehensive genotype–phenotype correlations provide valuable insights for personalized treatment strategies in type 2 VWD.
